# Calcium Chelation by Phosphate Ions and Its Influence on Fouling Mechanisms of Whey Protein Solutions in a Plate Heat Exchanger

**DOI:** 10.3390/foods10020259

**Published:** 2021-01-27

**Authors:** Luisa A. Scudeller, Pascal Blanpain-Avet, Thierry Six, Séverine Bellayer, Maude Jimenez, Thomas Croguennec, Christophe André, Guillaume Delaplace

**Affiliations:** 1 UMR 8207—UMET—Unité Matériaux et Transformations, INRAE, CNRS, Univ. Lille, Centrale Lille, 59000 Lille, France; pascal.blanpain-avet@inrae.fr (P.B.-A.); thierry.six@inrae.fr (T.S.); severine.bellayer@ensc-lille.fr (S.B.); maude.jimenez@univ-lille1.fr (M.J.); christophe.andre@yncrea.fr (C.A.); guillaume.delaplace@inrae.fr (G.D.); 2 Science et Technologie du lait et de l’oeuf (STLO)—UMR 1253, INRAE, Institut Agro, 35042 Rennes, France; thomas.croguennec@agrocampus-ouest.fr; 3 Laboratoire de Génie des Procédés, HEI, UC Lille, 59046 Lille, France

**Keywords:** fouling mechanism, whey proteins, calcium chelator, plate heat exchanger

## Abstract

Fouling of plate heat exchangers (PHEs) is a recurring problem when pasteurizing whey protein solutions. As Ca^2+^ is involved in denaturation/aggregation mechanisms of whey proteins, the use of calcium chelators seems to be a way to reduce the fouling of PHEs. Unfortunately, in depth studies investigating the changes of the whey protein fouling mechanism in the presence of calcium chelators are scarce. To improve our knowledge, reconstituted whey protein isolate (WPI) solutions were prepared with increasing amounts of phosphate, expressed in phosphorus (P). The fouling experiments were performed on a pilot-scale PHE, while monitoring the evolution of the pressure drop and heat transfer coefficient. The final deposit mass distribution and structure of the fouling layers were investigated, as well as the whey protein denaturation kinetics. Results suggest the existence of two different fouling mechanisms taking place, depending on the added P concentration in WPI solutions. For added P concentrations lower or equal to 20 mg/L, a spongy fouling layer consists of unfolded protein strands bound by available Ca^2+^. When the added P concentration is higher than 20 mg/L, a heterogeneously distributed fouling layer formed of calcium phosphate clusters covered by proteins in an arborescence structure is observed.

## 1. Introduction

Fouling of heat exchangers is a severe issue in the dairy industry as it is responsible for almost 80% of total production costs [1], as a result of supplementary energy input, loss of productivity, need for additional equipment and manpower, and everyday cleaning with harsh chemicals that increase environmental concerns [2,3].

Milk fouling of heat plate equipment appears during indirect ultra-high temperature (UHT), but also pasteurization treatments. For the latter, the fouling layer is composed of 50–70 wt% proteins, mainly β-lactoglobulin (β-lg), 30–40 wt% minerals (calcium, phosphorus, etc.) and 4–8 wt% fat [4].

Protein denaturation and salt precipitation (calcium phosphate [5], calcium carbonate [6], or calcium phosphate–protein/fat complexes, depending on the physical–chemical composition of dairy protein solutions) have been identified as the main underlying mechanisms of fouling. However, the exact pathways by which proteins and calcium salts interact and feed the layer of deposit on stainless steel surfaces are far from wholly understood, particularly for milk.

Moreover, the presence of casein micelles in milk complexifies the possibilities of protein aggregation and salt precipitation. Indeed, they constitute a source of proteins (alpha-, beta-, kappa-caseins) and minerals (through their calcium phosphate nanoclusters [7]), which can also modify the calcium balance between colloidal and soluble phases.

The understanding of mineral/protein interaction is better in whey, which could be considered as a simpler protein solution than milk due to the absence of casein micelles. Indeed, ionic calcium is assumed to be the binding agent of the whey proteins in the fouling layer recovered on the hot surface of the heat exchangers. Khaldi et al. [8] demonstrated that the calcium/ β-lactoglobulin (β-lg) protein molar ratio was a key parameter that changes the fouling layer structure. At low calcium/protein molar ratio, the fouling layer is thin and dense in which protein–protein interactions (here protein involved is β-lg) occur in majority. In contrast, at higher calcium/protein molar ratio, the structure of the deposit layer changes to a thicker and a looser structure, indicating that free ionic calcium content participates actively in the formation of the fouling layer.

As early proposed by Daufin et al. [5], calcium–protein interactions are probably initiated at a molecular scale through the carboxylate groups (aspartate, glutamate) of the whey proteins. As a consequence, fouling deposit at the surface of the exchanger plates can be considered, resulting from protein heat-unfolding and their aggregation by disulfide interchange reactions (-SH/SS), hydrophobic interactions, but also electrostatic shielding [9], and perhaps specific interaction between unfolded protein and ionic calcium (Ca^2+^), as indicated recently by Peixoto et al. [10]. To sum up, heating whey protein isolate (WPI) in the presence of calcium promotes a molecular link of unfolded proteins and allows the formation of protein-Ca^2+^-protein bridges, which facilitate the deposit build-up [11,12,13].

Due to the considerable importance of Ca^2+^ in the fouling layer formation, one possible way to reduce fouling is to reduce the amount of Ca^2+^ by prior addition of calcium chelators, such as phosphate and citrate salts to the solution.

The addition of calcium chelators is largely studied in milk solutions to promote functional dairy properties and to reduce protein aggregate formation in concentrated milk [14,15,16,17,18,19,20]. The effects of calcium chelators to reduce fouling have not been investigated in depth in the literature, especially those aiming at identifying the impact of calcium chelators on the fouling mechanisms. A brief sum-up of the major contributions that partially tackle this issue in whey proteins are, however, given below.

Hebishy et al. [13] observed on a lab-scale fouling ring that trisodium citrate (TSC), tripotassium citrate (TPC) and disodium hydrogen phosphate (DSHP) addition improves heat transfer during thermal processing. Decreasing the ionic calcium (Ca^2+^) concentration resulted in a decrease of the final solution viscosity and fouling after heat treatment at 95 °C for 2 min. However, in this study, the feed in proteins was ensured by a closed loop system, which means that the rate of native proteins entering the heat exchanger was not constant during the duration of the run. Such a system does not correspond to industrial practices.

Christian et al. [21] studied the fouling of the pilot-scale heat exchanger by whey protein concentrates in the presence of calcium chelators. Unfortunately, they simultaneously changed the amount of calcium and phosphorus-containing compounds, which prevents concluding which mineral species is responsible for the decrease of fouling, and which phenomena governs the changes in the deposit composition. Note that Christian et al. [21] did not measure experimentally the decrease of deposit mass weight with chelator addition, but they reported the change in the dynamics of the fouling phenomena by monitoring the pressure drop and thermal resistance.

Finally, the sole work that shows, at a pilot scale, the influence of calcium chelators by fixing the amount of protein and Ca^2+^, and changing only the phosphate concentration, is that of Blanpain-Avet et al. [22]. They followed the decrease of the fouling deposit mass when phosphate was added in the system. More importantly, the two major descriptors used in the industry (namely the pressure drop and the thermal resistance values) were not given and, consequently, it is not possible to compare the dynamic of the evolution of these parameters in respect of deposit mass decrease. Nevertheless, Blanpain-Avet et al. [22] were also the first to report electron probe micro analyses (EPMA) of the fouling layer and attempted to provide a molecular scale interpretation of how the structure of the deposit is modified with the increasing calcium chelator concentration.

To sum up, the analysis of the bibliography reveals that few contributions address the effects of calcium chelators on fouling by dairy derivatives. Moreover, among the studies listed above, works are led (i) at different scales (laboratory and pilot), (ii) with different dairy derivatives (milk or whey protein solutions), and iii) use various physical variables to quantify fouling (deposit mass, pressure drop, increase in thermal resistance). That is why it is difficult to get a clear overview about the underlying fouling mechanism in the presence of calcium chelators. Moreover, kinetics studies dealing with the influence of calcium chelators on β-lg denaturation are absent.

Consequently, it is impossible to precise, at this stage, whether calcium chelators addition (i) only deplete the media of the required binding agent, Ca^2+^, for fouling layer growth, or (ii) also modify whey protein denaturation reaction and lead to lower amounts of unfolded proteins to form the deposit layer.

The objective of the present study is to determine the role of DSHP, both at molecular and pilot scales, on the mechanism of fouling of the plate heat exchangers by calcium-containing whey protein solutions. For this, whey protein isolate solutions at 0.5 wt% of powder containing fixed calcium content and various amounts of phosphate were used to conduct the fouling experiments at a pilot scale using a plate heat exchanger (PHE). Continuous monitoring of the pressure drop and heat transfer coefficient and the amount of deposited fouling mass along the PHE after two hours was carried out. In parallel, the β-lg denaturation kinetics of some of the fouling solutions were investigated and discussed. EPMA X-ray mappings were also performed and innovative overlay representations were used to analyze evolution of WPI fouling layer structure with added phosphate concentration.

This study focused on the understanding of fouling of the PHE by whey protein solutions, having different levels of free Ca by adding phosphate as a calcium chelator. The nutritional effect of adding phosphate in the whey protein solution was secondary in this paper, even if this question is essential when dealing with dairy products. Indeed, phosphorus is only one chelating agent among many possibilities to investigate fouling phenomena in absence, or reduce quantity of calcium under its simplest ionic form. From a nutritional point of view, we should mention that the formation of calcium phosphate (CaP) will only reduce the amount of Ca^2+^ in the milk, but for human consumption, the Ca is still present in the form of CaP. Moreover, even if our concern was, not at all, the question of nutrition, we paid attention in order that the amount of added phosphorus (max 50 mg/L) was within the legal limit (1500 mg/kg), as defined by the Codex Alimentarius (CODEX STAN 192-1995 [23]).

## 2. Materials and Methods

### 2.1. Model Fouling Solution

WPI is a Prolacta 95 powder supplied by Lactalis ingredients (F-35230 Bourgbarré, France). The powder is composed by 90% proteins, 0.4% fat, 3% lactose, 3% ash, and 6% moisture. β-lg and α-lactalbumin are the 2 major whey proteins, but β-lg is the main protein responsible for fouling in milk and milk protein derivatives [1,24], so the emphasis of this work will be on the β-lg behavior.

WPI solution was reconstituted at powder concentration of 0.5% (*w*/*v*) in the launching tank using reverse osmosis water. Powder dispersion occurred for at least two hours by gently stirring the solution (this also allows the complete rehydration of β-lg molecules). Total β-lg concentration ([β-lg]) was 3.05 ± 0.15 g/L on average as determined by reverse phase HPLC (see below).

Known amounts of anhydrous CaCl_2_ (96%, Acros Organics, Thermo Fisher Scientific, Waltham, MA, USA) and of disodium hydrogen phosphate (anhydrous Na_2_HPO_4_, 99.0% purity, supplied from Sigma Aldrich, Saint-Louis, MO, USA) were separately dissolved in a fraction of the water taken from the launching tank. Salts were dissolved at room temperature with a constant stirring, for 1.5 h. Then, the phosphorus and calcium solutions were mixed with the rest of the protein solution and the mixture was let under stirring for 0.5 h before the fouling test began.

The amount of CaCl_2_ was targeted to have 100 mg/L of Ca in the final solution as it was established that this level allowed (i) enough fouling of the PHE after two hours (without plugging), and (ii) to compare and to go further than the work of Blanpain-Avet et al. [22]. Average total calcium concentration ([Ca]) in WPI solutions was 101.02 ± 2.06 mg/L, determined by atomic absorption spectrometry using a Spectro AA 55B apparatus, (Varian, Palo Alto, CA, USA) as [22].

### 2.2. Experimental Fouling Installation

**Figure 1:**
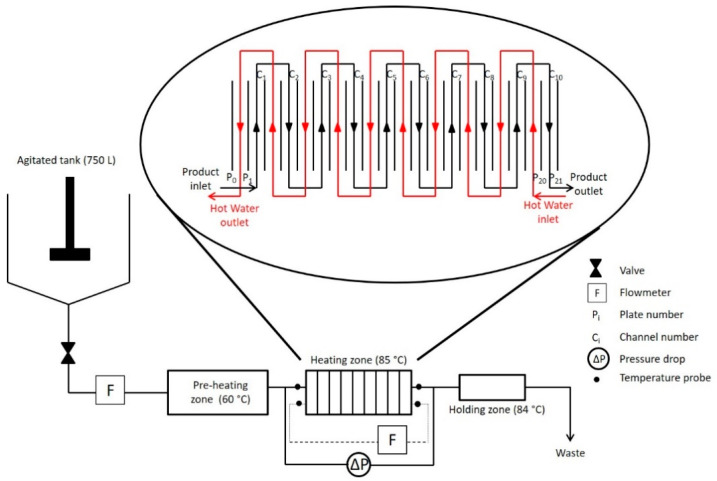
Schematic diagram of the experimental set-up with the plate heat exchanger flow arrangement to study fouling behavior of 0.5% whey protein isolate (WPI) solution as a model fluid.

Pilot scale fouling experiments were conducted, as previously described [8,22,25], in a plate heat exchanger represented schematically in Figure 1. Fouling installation was divided in two distinct zones: a pre-heating and a heating zone composed of a PHE (VICARB, model V7, Alfa-Laval, Saint-Priest, France). The PHE was used in a countercurrent configuration, consisting of 21 V7 corrugated plates (35° corrugation angle) forming ten passes of one channel for the two sides. The space between two plates (e) was 4.1 mm and the total channel exchange surface was 1.5 m^2^.

The WPI solution passed through the preheating zone to ensure the required inlet temperature in the heating zone of 60 °C. Once in the heating zone, the product temperature increased using hot water that was adjusted to ensure a constant outlet temperature of 85 °C. Both product and hot water flow rates were 300 L/h (corresponding to a Reynolds of 2800 calculated according Equation (1) for the entire experiment duration (2 h). The WPI solution was not recirculated as in industrial practice.

The average Reynolds number, *Re*, along the PHE was determined at a clean state from the Equation (1):

(1) $$Re=\frac{\rho \;v\;D_{h}}{\mu }=\frac{2\;\rho \;Q}{\mu \;l},$$

where ρ is the fouling solution density in kg/m^3^, v is the average fluid velocity in m/s (which is also equal to *Q/l*·*e*), *Q* is the fouling solution flow rate in m^3^/s, l is the plate width in m, *µ* is the fouling solution viscosity in Pa.s, and *D*_h_, in m, is the hydraulic diameter defined by: *D*_h_ = 2·*e* as classically encountered in other publications [22,26,27,28]. The water physical properties at the mean temperature in the PHE were used to calculate Re once the presence of a 0.5 wt% WPI in water does not modify them significantly, as assumed by [22,25,26,29]. Indeed, Petit et al. [27] estimated the density of the WPI at 1025 kg/m^3^ while the true water density at 20 °C is 996 kg/m^3^, so the approximation of volume additivity due to the 0.5% WPI leads to 996.1 kg/m^3^ which is almost the water density. For this powder concentration, the protein concentration is not high and viscosity is expected to be close to the water one, even in the presence of β-lg aggregates.

The Reynolds number in the PHE calculated for the fouling runs was 2800, and corresponded to a turbulent regime. Indeed, Leuliet [28] established for the corrugated V7 plates used in this work that the turbulence regime occurs over *Re* = 260.

### 2.3. Fouling Mass

All of the numbered plates were previously weighed (error of ±0.1 g) and after each fouling test (duration of fouling run of two hours) the heating zone was opened to let the plates lose their excess water. The next day, all of the plates were put in an oven at 50 °C until all of the fouling was completely dried. The plates were then weighed at room temperature and the dry deposit mass on each plate was calculated by the difference of the mass before and after the fouling experiment. Fouling mass in a single channel was obtained by adding the two deposited masses obtained for the plates constituting the channel wall. The experiment was repeated twice for each phosphorous concentration.

### 2.4. Monitoring of the Heat Transfer Coefficient and Pressure Drop in the Plate Heat Exchanger

Experimental data measurements were made using the following sensors: (i) magnetic induced flowmeters (Krohne IFM 6080 K, Krohne, Romans-sur-Isère, France) for the flow rates, (ii) platinum resistance probes (PT100) for temperatures of all fluids, and (iii) transmitter for differential pressure (Siemens 7MF4433, Siemens, Munich, Germany) for the PHE pressure drop. All parameters were collected via a data acquisition system (Agilent Technologies, Santa Clara, CA, USA) with an acquisition period of 5 s.

The pressure drop, Δ*P*, evolution with time in the heating zone of the PHE was measured to evaluate the amount of fouling in the system. Physically, as fouling increases, the fouling layer becomes thicker, the cross section of each channel of the heat exchangers narrows, and the measured value of pressure drop along the PHE increase.

The set of dimensionless numbers conventionally used to describe the hydrodynamic behavior of a PHE are Reynolds numbers and friction factor, *f*/2, defined as:

(2) $$\frac{f}{2}=\frac{\Delta P\;D_{h}}{4\;L_{tot}\;\rho \;v^{2}},$$

where *L_tot_* is the length of the path achieved by the fouling fluid within PHE. Replacing as in Reynolds number v by *Q*/*l*·*e* and *D_h_* by 2·*e*, the following relation is found:

(3) $$\frac{f}{2}=\frac{\Delta P\;l^{2}\;e^{3}}{2\;L_{tot}\;\rho \;Q^{2}}.$$

Leuliet [28] measured the influence of Reynold number on the friction factor and proposed an empirical correlation describing the evolution of friction factor for each flow regime. For the turbulent regime, he proposed the Equation (3):

(4) $$\frac{f}{2}=\frac{1.6225}{Re^{0.163}}.$$

As *Re* is constant during the run, *f*/2 will also be constant and, consequently, Δ*P* will always be proportional to *e*^−3^, with e being the space between two plates. Therefore, it can be immediately noted that (i) pressure drop monitoring is sensitive to evaluate the variation of the channels cross section due to fouling (ii) pressure drop along the PHE is a global measure, which is also limited, as it cannot accurately describe the local distribution of fouling layer, responsible of the decrease of the space between two plates.

Similarly, the transfer of heat from the calorific fluid (also named hot fluid) to the product (also named cold fluid) is more difficult when the fouling layer is thicker. Indeed, the thermal conductivity of the protein deposit is lower than that of steel (16 W·m^−1^·K^−1^ for the steel versus 0.1–0.5 W·m^−1^·K^−1^ for the protein deposit [30]). Consequently, when the protein layer forms, it is necessary to deliver more heat to keep the outlet temperature of the product constant at 85 °C. To monitor the fouling growth, the decrease of heat transfer coefficient, *U* (W·m^−2^·K^−1^) with fouling run time or the increase of thermal resistance (1/*U*) was calculated from the energy balance in the heat exchangers:

(5) $$U=\;\frac{Q_{p}}{S\;\Delta T_{LM}},$$

where: *S*—total exchange surface in the heating zone (m^2^); Δ*T_LM_*—logarithmic mean temperature difference (K) expressed as:

(6) $$\frac{\left(T_{wi}-T_{po}\right)-\left(T_{wo}-T_{pi}\right)}{\ln\left(\frac{T_{wi}-T_{po}}{T_{wo}-T_{pi}}\right)},$$

where *T_po_* and *T_pi_* are the outlet and inlet temperature (K) of the product, respectively, and *T_wo_* and *T_wi_* are the outlet and inlet temperature (K) of the hot water, respectively; *Q_p_*—amount of heat (W) exchanged during the heat treatment of the product; it is calculated by:

(7) $$Q_{p}={\dot{m}}_{p}\;c_{p}\;\left(T_{po}-T_{pi}\right),$$

where *C_p_* is the product heat capacity (J·kg^−1^·K^−1^) and ${\dot{m}}_{p}$ is the mass flow rate of the product (kg·s^−1^) equal to the product of volumetric flow rate, *Q* (m^3^·s^−1^) by the density *ρ* (kg·m^−3^) of WPI solution.

For the powder concentration used 0.5% (*w*/*v*), the protein concentration is not high and specific heat capacity is also expected to be close to the water one, even in the presence of β-lg aggregates [29].

### 2.5. β-Lg Denaturation Kinetics Determination

All thermal denaturation experiments were conducted on twelve stainless steel tube of 1.5 mL for 3 different phosphorous concentrations (0, 10, and 50 mg/L of *P*). The WPI samples were preheated at 60–65 °C, below the β-lg denaturation temperature of 77 °C [31,32] in a first water bath to shorten the time to reach the desired holding temperature. Then, the samples were immersed in a second water bath, where temperature was maintained 10 °C higher than the desired holding temperature. The holding temperature range was 70–92 °C. When the desired holding temperature was reached in the second water bath, the sample was transferred in a third water bath, where the temperature was fixed 2 °C higher than the desired holding temperature. The immersion of the WPI sample in this last water bath corresponded to time zero of the heat denaturation kinetics. The samples were removed at different times and immediately cooled in a beaker filled with ice to stop the denaturation process as described in Khaldi et al. [30].

### 2.6. HPLC Analysis

The native β-Lg concentration after heat treatment was determined by HPLC using the supernatant recovered after centrifugation (9000 rpm for 30 min at 4 °C) of the samples adjusted to pH 4.6. The chromatographic system was an Alliance e2695 Separation Module (Waters, Milford, MA, USA), composed by a XBridge Protein BEH C4 300 Å, 3.5 µm, 4.6 mm × 250 mm separation column associated with a guard column (Sentry Guard Cartridge, Waters). The two mobile phases used were 0.1% (*v*/*v*) of trifluoroacetic acid (99%, Acros Organics) in Milli-Q water as solvent A and 0.1% (*v*/*v*) trifluoroacetic acid, 80% acetonitrile (HPLC grade, Thermo Fisher Scientific) and 20% Milli-Q as solvent B. 20 µL of each sample was injected in the column maintained at 40 °C. The separation was performed at a flow rate of 1 mL/min with gradient elution and a detection wavelength of 215 nm. Analyses were repeated two times for each sample and three times for each standard. Calibration standards in the range from 0.25 to 3 g/L were prepared by dissolving a β-lg powder (BioPure industrial powder, Davisco, Foods International, Inc., Minnesota: β-lg at 88.85% purity) in Milli-Q water.

The native β-lg concentrations were calculated by averaging the measured chromatographic areas and converting each area into concentration by means of the calibration curve.

### 2.7. Determination of β-Lg Kinetic Parameters

The reaction model used is based on an approach considering the conversion of the soluble (native and unfolded proteins) into insoluble (aggregated proteins) species as in [33,34] and other work of our groups [25,26,27,30,35]. This transformation is defined as:

(8) $$-\frac{dC}{dt}=k_{n}C^{n},$$

where *C* is the soluble β-lg concentration (g/L), *k_n_* the denaturation rate constant for a reaction order equal to *n* (g^1-n^·L^n−1^·s^−1^) and *t* is the time (s). For this work, *n* was taken as 1.5 as in others works of our groups [8,26,27,30,35] and the solution of the denaturation equation gives:

(9) $$k_{1.5}.\;t=\frac{\sqrt{\frac{C_{o}}{C\left(t\right)}}-1}{0.5\;\sqrt{C_{o}}},$$

where *C_o_* is the initial soluble β-lg concentration (time zero).

For a narrow range of temperature, the relation between the denaturation rate constant (*k*_n=1.5_) and the holding heat treatment temperature (T) can be described by an Arrhenius equation:

(10) $$\ln\left(k_{1.5}\right)=\ln\left(k_{1.5}^{o}\right)-\frac{E_{A}}{R\;T},$$

where $k_{1.5}^{o}$ is the frequency factor (g^−0.5^·L^0.5^·s^−1^), *E*_A_ is the activation energy (J/mol), *R* is the universal gas constant (~8.314 J·K^−1^·mol^−1^), and *T* is the temperature (K).

### 2.8. Electron-Probe Micro Analysis (EPMA)

For EPMA analysis, 316L stainless steel plates, of dimension 15 × 10 × 1 mm^3^, were put in the holding zone. After fouling experiments, the fouled plates were dehydrated by the critical point method in an E300 Critical Point Dryer (Quorum Technologies Ltd, Laughton, England) in order to keep the original structure that could change with the oven drying process. Once dried, samples were embedded into epoxy resin, polished, and carbon coated with a Bal-Tec SCD005 sputter coater (Leica, Nanterre, France). Cross-section chemical characterizations of fouled samples were obtained using a Cameca SX100 EPMA (Cameca, Paris, France) through elemental analysis. X-ray mappings were carried out at 15 kV, 40 nA. For the mappings, the crystal used to detect the Kα of S (characteristic of protein deposit) and Ca (characteristic of Ca deposit) was PET, the crystal used to detect the Kα of P (characteristic of P deposit) was the TAP and the crystal used to detect the Kα of Fe (characteristic of the substrate) was a LiF.

Similarly to recent publication of our group [7] studying the composition of fouling layer induced by heat treatment of casein/WPI solutions, an overlay of the analyzed elements (Ca, P, S, Fe) was performed to identify the presence or not of co-localized elements. The aim of this image processing was to check whether CaP clusters gradually appears in the fouling structure when P concentration increases, which could help clarifying the change in mechanism of fouling formation induced by P addition in WPI solutions.

## 3. Results and Discussions

### 3.1. Effect of Adding Phosphate on the Deposit Mass Distribution within the PHE and on Total Deposit Mass, and Highlight the Role of Free Calcium on Fouling Mechanism

#### 3.1.1. Total Deposit Mass

**Figure 2:**
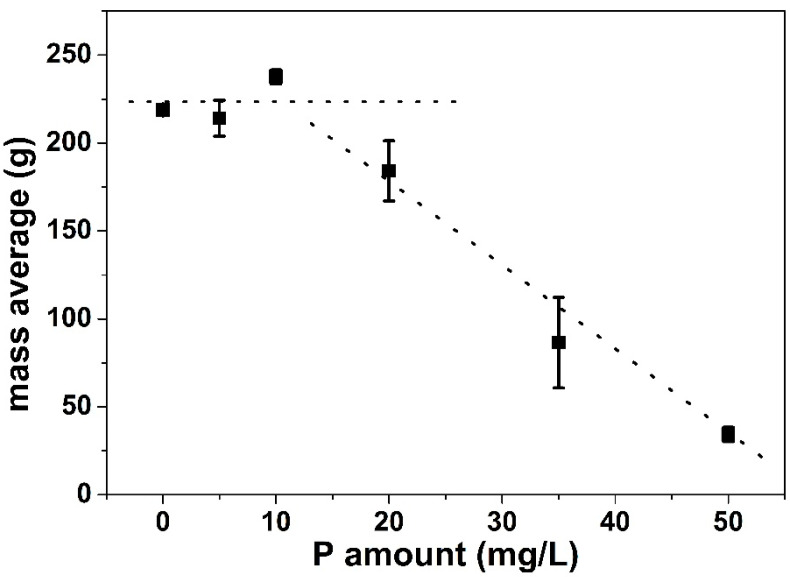
Variation of the measured total deposited mass within the plate heat exchanger (PHE) as a function of the added P at an average calcium concentration equal to <[Ca]> = 101.02 ± 2.06 mg/L and 0.5% (*w*/*v*) (WPI) solution as a model fluid. Dotted lines are given for guiding eyes.

Figure 2 represents the mean total dry mass (g) deposited in the PHE (average of two runs for each condition) in function of the amount, in mg/L, of phosphorus added (from phosphates) in the WPI solutions. The amounts of Ca and β-lg in the WPI solutions were constant and the average values measured by atomic absorption and HPLC were equal to [Ca] = 101.02 ± 2.06 mg/L and [β-lg] = 3.05 ± 0.15 g/L, respectively. Consequently, the Ca/β-lg molar ratio in the fouling solutions was 15.

According to Figure 2, it can be observed that the total mass of deposit did not change significantly, compared to a solution free of phosphorus, after adding 5 and 10 mg/L of P in the solution. This plateau value was followed by a decrease of total deposit mass when 20 mg/L of P is added in the WPI solutions. When the P concentration continues to increase, the total deposit mass becomes low (~30 g for ten channels after adding 50 mg/L of phosphorus). It was decided to limit phosphorus additions up to 50 mg/L because, beyond this amount, it was difficult to measure the mass of deposit on individual plate.

The decrease of the total deposit mass after adding calcium chelators to the 0.5% (*w*/*v*) WPI solutions is in agreement with the work of Hebishy et al. [13]. These authors reported that, in a laboratory scale fouling ring, the fouling potential of a WPI solution at a concentration of 3.0% (*w*/*v*) was prevented with trisodium citrate, tripotassium citrate, or disodium hydrogen phosphate. Results are also coherent with those of Christian et al. [21] working with WPC solutions at pilot scale. They showed that doubling the quantity of calcium and phosphorus of a 0.3 wt% β-lg solution decreased the extent of fouling. However, they changed Ca and P content simultaneously so further comparisons with the present work is difficult. In addition, the heat treatment of the protein solution was carried out in a close loop so depletion of protein and mineral exist during the fouling runs, which was not the case in the present study.

Finally, our work was compared with that of Blanpain-Avet et al. [22]. The same trend was observed concerning the decrease of total deposit mass with increasing P concentration. The only difference between Blanpain-Avet et al. [22] work and this study is that, for the former, the deposit mass decreases instantly after phosphorus addition in the WPI solution, while for the latter, the decrease of the total mass of fouling is delayed and started only after 20 mg/L of P is added.

One possible explanation regarding such difference could be the variability of the protein powder batches between these two works. These two batches do not have the exact same β-lg amount, resulting in different values of calcium/β-lg molar ratio when the fouling solutions were prepared using similar powder concentrations and calcium content. For this work, the measured value of calcium/β-lg molar ratio was 15 instead of 16 for Blanpain-Avet et al. [22], but this slight difference hardly explains the different results obtained. As we have not evaluated all of the powder compounds (protein, mineral, fat, lactose, etc.) and we did not master all production steps of this commercial powder, it is difficult to discuss further this aspect.

Note that, from a chemical point of view, the fact that the total deposit mass does not fall immediately after the addition of P appears rationale. Indeed, in literature, it is admitted that each unfolded β-lg can bind up to three calcium ions [10,36,37]. The calcium/β-lg molar ratio in the WPI solution (15) is well beyond the required calcium/β-lg molar ratio to let the fouling mechanism happen and, consequently, it seems logical that a significant concentration of added P is required before the amount of Ca^2+^ is sufficiently reduced to decrease the fouling potential of unfolded β-lg.

Below 20 mg/L of P addition in the WPI solutions, the added P probably complexes Ca^2+^ in excess and consequently the Ca^2+^ content in the vicinity of the protein stayed high enough to promote fouling. Above this added P threshold value (20 mg/L), calcium chelation significantly reduced the level of Ca^2+^ near the protein leading to a change in the fouling mechanism and to a significant decrease of the total deposit mass.

**Figure 3:**
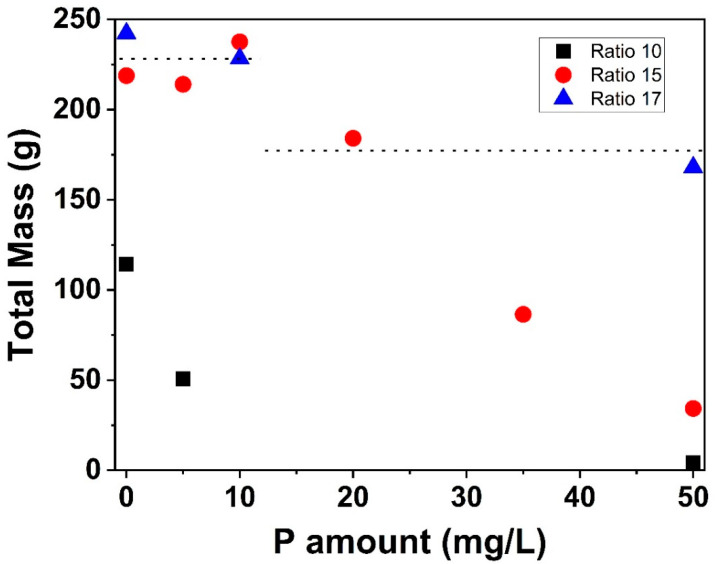
Variation of the measured total deposited mass within the PHE as a function of the added P for a 0.5% (*w*/*v*) WPI solution and three different Ca/β-lactoglobulin (β-lg) molar ratio as a model fluid. The square is for ratio 10, circle for ratio 15, and triangle for ratio 17. Dotted lines are given for guiding eyes.

Finally, to check whether more P is required to reduce fouling when Ca/β-lg molar ratio increases due to calcium increase, further fouling runs were carried out with different Ca/β-lg molar ratio (Figure 3). The total protein content was kept the same (0.5% *w*/*v*), changing only the final total Ca concentration, to have a Ca/β-lg molar ratio of 10 and 17.

Without added P, the total fouling mass was 120 g for WPI solution with Ca/β-lg molar ratio of 10, almost half of the total mass observed for Ca/β-lg molar ratio of 15 (220 g) while for Ca/β-lg molar ratio of 17, this value was equal to 240 g. These results highlighted again that, in absence of P, a change in Ca content impacts strongly fouling mechanism of WPI solutions as previously mentioned in literature [8,9,10,11,12,13].

As soon as P was added in the WPI solution with Ca/β-lg molar ratio of 10, the total fouling mass started to decrease. On the contrary, when Ca/β-lg molar ratio increases (15 and 17), a plateau was formed, indicating that a higher P concentration is needed to start to reduce fouling. It can be also observed on Figure 3 that the required quantity of added P to initiate fouling mass deposit reduction is much more important when Ca/β-lg molar ratio increases. In fact, to obtain almost the same fouling mass reduction, 50 mg/L of added P are necessary for the solution having a Ca/β-lg molar ratio of 17, while 20 mg/L of added P are needed for the solution with Ca/β-lg molar ratio of 15.

#### 3.1.2. Deposit Mass Distribution

**Figure 4:**
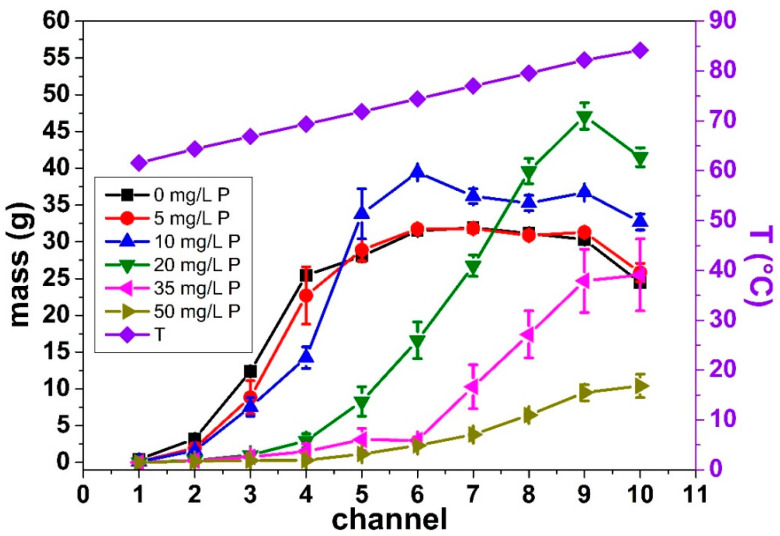
Dry deposit mass per channel along the PHE for all P concentrations and the temperature profile in the heating zone. The model fluid was a 0.5% (*w*/*v*) WPI solution with an average calcium concentration equal to <[Ca]> = 101.02 ± 2.06 mg/L.

Further analysis shows the total deposit mass changed with increasing P content in the WPI solution, as well as the mass distribution within the PHE (Figure 4).

Figure 4 illustrates the evolution of deposit mass per channel for the fouling solution having a Ca/β-lg molar ratio of 15. Each symbol corresponds to different added P content (mean value computed for two similar runs) and the temperature reached by the fouling solution in each channel is plotted on right ordinate axis.

No change of fouling mass distribution in the PHE was observed for low P concentration in the WPI solutions (5 and 10 mg/L), compared to the condition without added P (0 mg/L). For this first group of fouling runs (0, 5, and 10 mg/L), the fouling started in the second channel, and then sharply increased until it reached a maximum value (~30 g) in the fifth channel. The small mass for the first channels is normal, once below 75 °C, the level of β-lg denaturation is low with small and reversible van der Waals bonds formed between proteins; while above 75 °C, the denaturation level is high with irreversible covalent disulfide bonds formed [12,38].

For higher P content (>10 mg/L) in the WPI solutions, the observed mass distribution profile was different from those of the first group. It seems that the first channel for which the fouling appears is delayed (that is, at a higher temperature, Figure 4). Moreover, the shape of mass distribution clearly changed, as no plateau value for deposit mass is clearly identified, as it was the case when P concentration is inferior to 10 mg/L. The reduction of the fouling in the first channels when the added P concentration increased is in agreement with the literature, because, as was reported, in lab scale, the calcium-binding agents increase the heat stability of the WPI solutions and, consequently, reduce protein aggregation [11,13,14,15]. This is due to the presence of phosphate, which reduces the amount of free calcium limiting the deposition of protein on the surface of the heat exchanger, delaying its appearance at higher temperature.

It can also be noted that the maximum value for deposit mass can sometimes become higher than the maximum value reported in absence of P (P = 10 mg/L and P = 20 mg/L). Despite the lack of a clear explanation about this behavior, one possible explication is that the phosphorus increases the heat stability of the solution. With the increase of the WPI solution heat stability, the β-lg will unfold latter, i.e., at high temperature (last channels). In these channels, there will be more proteins available to form the fouling layer than in experiments conducted at lower added P concentrations (<20 mg/L).

This difference in the mass distribution within the PHE was already observed by Blanpain-Avet et al. [22]. Indeed, they also showed that without phosphorus, fouling starts in the first channel increasing quasi-exponentially until the 5th channel, and then remained constant, as this is the case here for low added P concentration (<20 mg/L). In their study, as soon as phosphorus was added, fouling was reduced, also changing the mass distribution profile. In the presence of P, the onset of deposit build-up was shifted to a higher temperature. The amount of deposit located at the last channel (10th) was a decreasing function of added P, as observed in our case for added P concentration larger than 10 mg/L.

### 3.2. Effect of Adding Phosphate on the Pressure Drop, on the Heat Transfer, and on the Fouling Layer Structure, and Highlight on Fouling Build-Up in Function of Calcium Complexes

#### 3.2.1. Pressure Drop

**Figure 5:**
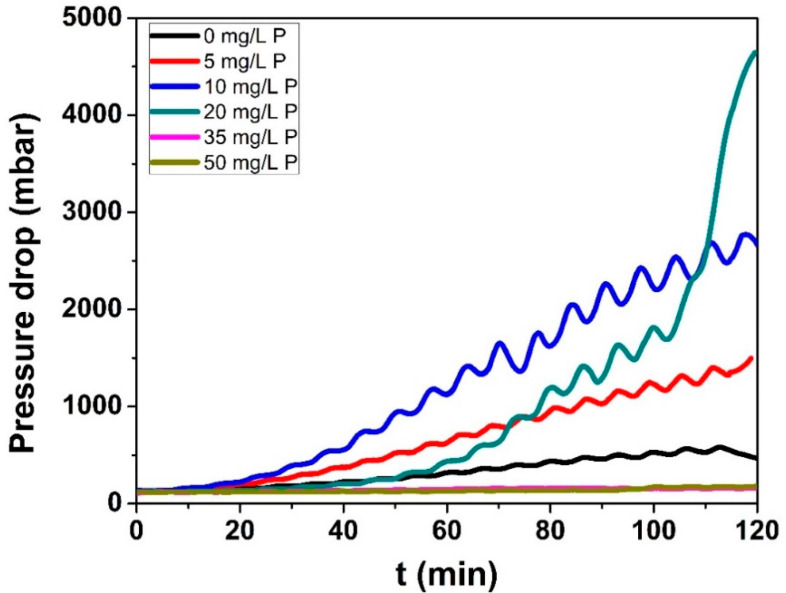
Pressure drop during fouling experiment for all phosphorus concentration in a 0.5% (*w*/*v*) WPI solution with a calcium concentration equal to <[Ca]> = 101.02 ± 2.06 mg/L.

Figure 5 shows the pressure drop evolution during fouling experiments for the WPI solutions with a Ca/β-lg molar ratio of 15 for all of the added P concentrations. This is a global measure made on the whole fouling zone in a turbulent regime, as explained previously in the Material and Methods section. Even if this measure is not perfect, since, in the PHE, the fouling layer is not homogenously deposited within a channel and along a channel due to the existence of the temperature profile along the heat exchanger, it is expected that the thicker the fouling layer, the smaller the channel cross section and the higher the pressure drop.

As was previously reported for the total mass and mass distribution, the pressure drop also shows two different behaviors, according to the added P concentration.

For concentrations of added P smaller or equal to 20 mg/L, an increase in pressure drop with a maximal pressure drop for 20 mg/L of added P is observed. This increase for added P smaller than 20 mg/L started during the first 20 min of the fouling experiment and kept increasing linearly until the end of the experiment. For 20 mg/L, the increase in the pressure drop was different, following the same behavior as without P during the first hour and then it increased abruptly, reaching a maximum value higher than that of 10 mg/L of added P (maximum fouling mass). It looks like a transition to a different pressure drop behavior. Indeed, for added P concentrations of 35 and 50 mg/L, the pressure drop almost did not change from their initial value.

These different evolutions of pressure drop with time in function of the amount of added P in the WPI solutions reinforce the fact that two different deposit mechanisms take place, depending on added P concentration.

Indeed, the total mass and mass distribution were the same for 0 to 10 mg/L of added P; however, the pressure drop significantly increased as the P content in the WPI solution increased up to 20 mg/L. Besides the pressure drop be proportional to fouling width, the limitation of pressure drop measurements for investigating fouling mass has been already mentioned. We cited Burton [39]: “quantitatively the pressure differences curves did not reflect sufficiently well the corresponding changes in the amount of deposit found on the plates at the end of experiments”. Nevertheless, for the case aforementioned, it is likely that the increase of pressure drop reveals a change in the structure of the fouling layer. Indeed, we will see later (Section 3.2.3) that the structure of the fouling layer is not exactly the same after addition of P, and appears slightly more voluminous.

For higher amounts of added P (35 and 50 mg/L), the pressure drop does not evolve with time and is kept unchanged from their initial values measured at a clean state, indicating that little fouling occurs. This lack of evolution for pressure drop seems time consistent with the visual observation made at the end of the fouling runs (not shown here), and measurements obtained for total deposit mass (Figure 2), showing a drastic decrease of the deposit mass when the added P concentration becomes higher than 20 mg/L.

#### 3.2.2. Heat Transfer

With this noticeable increase in the pressure drop (for runs with P concentration ≤20 mg/L), it is expected that more heat is required to maintain the outlet temperature of the fouling solution constant, since a layer of fouling deposit should form. To verify this assumption, the heat transfer coefficient for the WPI solutions with a Ca/β-lg molar ratio of 15 for all of the added P concentrations was calculated, Equation (6).

**Figure 6:**
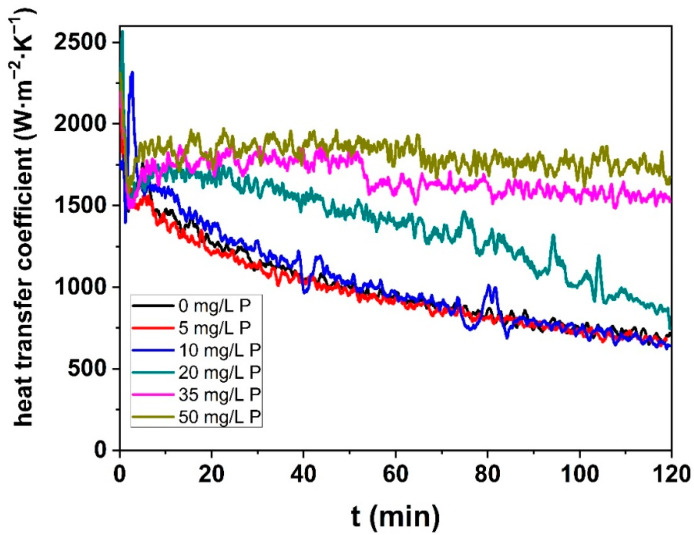
Heat transfer coefficient during fouling experiment for all phosphorus concentration in a 0.5% (*w*/*v*) WPI solution with a calcium concentration equal to <[Ca]> = 101.02 ± 2.06 mg/L.

The calculated heat transfer coefficient, Figure 6, also shows two different behaviors (0–10 mg/L and 35–50 mg/L), with a transition state observed for 20 mg/L. For low added P content (≤20 mg/L) in the WPI solutions, the heat transfer coefficient decreases. For 20 mg/L, the heat transfer reached almost the same final value than those for solutions with low added P concentration (<20 mg/L), but its profile decreased differently, indicating again a transition behavior. In contrast, for 35 and 50 mg/L of added P in the WPI solutions, the heat transfer coefficients almost remain constant during the run.

The evolution of heat transfer coefficient with time are superimposed for the added P concentrations (0, 5, and 10 mg/L) indicating that the product of thermal properties and mean thickness of the fouling layer follows almost the same trends for these three fouling runs.

These results for heat transfer coefficient are in accordance with the fact that similar total fouling mass were measured for these three fouling runs. However, these results disagree with the pressure drop tendency (Figure 5), which increase for this range of added P concentration, indicating an increase of fouling layer. One possible explanation for this apparent discordance (for P <20 mg/L) is that as added P increases, a more spongy deposit might form (explaining the increase in pressure drop), but with a slightly different structure (more porous, less compact, etc.) that is less insulating, leading at the end to a similar heat transfer coefficient.

The evolution of a heat transfer coefficient with time for an added P concentration equal to 20 mg/L was not superimposed, with the curve obtained, when no P was added. Despite almost the same initial and final values than the curves for added P <20 mg/L, the profile of the decrease is different; so that for 20 mg/L the coefficient is always higher than for concentrations of add P <20 mg/L. This slow decrease indicates that the heat resistance due to the fouling deposit starts to change when added P reaches such a level of concentration. This transition behavior is logical since, when added P concentration is equal to 20 mg/L, the total fouling mass starts to go down, and as a result, it is expected that the insulating properties are reduced.

For concentrations higher than 20 mg/L of added P, the heat transfer coefficient does not evolve with time during the whole fouling experiment, in accordance with the absence of the pressure drop. This happens because the mass fouling deposited onto the surface of the PHE is significantly reduced (see Figure 4) and becomes weak and, consequently, the heat transfer coefficient is poorly affected during the thermal process.

#### 3.2.3. Fouling Layer Structure

**Figure 7:**
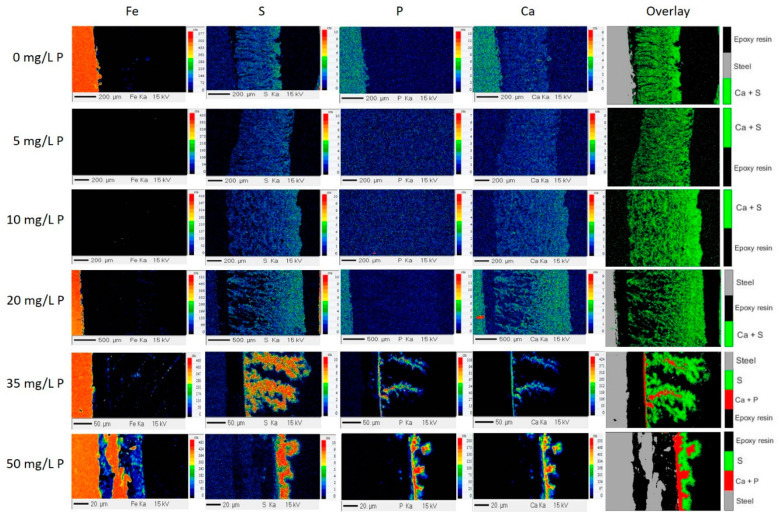
Fe, S, P, Ca, and the overlay X-ray mappings of the cross section of the fouling layer as a function of the added P in the WPI solutions containing 100 mg/L of Ca and 0.5% *w*/*v* of protein. For the samples 5 mg/L P and 10 mg/L P the fouling completely detached from the substrate.

Christian et al. [21] and Khaldi et al. [25] already suggested that the heat transfer coefficients varies with the structure of the deposit layer. To verify this statement and understand the influence of the phosphorus in the fouling building up, cross-section chemical characterization of fouling on the stainless-steel surface was carried out by the EPMA technique, Figure 7.

Two different structure of fouling layer (and hence fouling mechanisms) can be clearly identified according to the added P concentration in the WPI solution: (a) for added P concentrations lower or equal to 20 mg/L, and (b) for added P concentrations of 35 and 50 mg/L.

For added P concentrations lower or equal to 20 mg/L, the thickness of the deposit increases from ~430 µm to ~1800 µm with an increasing amount of phosphorus added in the WPI solution up to the concentration of 20 mg/L. For this range of P concentration, the fouling structure shows spaces near the substrate surface and then a densification. Moreover, no clusters of Ca or calcium phosphate (CaP) were evidenced. For all of these samples, the calcium and the protein are co-located, as seen on the overlay images in Figure 7.

Beyond 20 mg/L of added P, the fouling thickness decreases considerably and it is possible to see that phosphorous participates directly to the deposit layer, forming clusters of calcium phosphate that are embedded in the protein matrix (overlay images, Figure 7). From the overlay images, it is observed that a CaP layer was first formed on the steel surface and then the protein binds to this CaP structure. For such added P concentrations, CaP becomes the only binding agent for proteins, which accumulate around the CaP clusters.

For added P concentration up to 20 mg/L, it seems that P did not contribute directly in the fouling build-up, but it is expected that it decreased Ca^2+^ concentration by chelating the Ca^2+^ in the solution for forming CaP. Consequently, it probably decreased protein charge screening modifying the fouling structure due to the increased electrostatic repulsions between proteins. This phenomena is clearly more present near the steel surface, implying that the CaP are formed preferably close to the surface, thus changing the charge screening of proteins that initiate the fouling build-up.

Indeed, the deposit appears more heterogeneous and looser near the substrate and becomes denser when moving away from it. This can be observed clearly when analyzing S (representing protein) and Ca images when added P increases. This change of structure organization of the deposit close to the surface is assumed to be due to the appearance of small amount of CaP near the substrate surface. The gradual appearance of CaP cluster in solution and close to steel surface can also explain the increase of the fouling layer thickness, for concentration of added P up to 20 mg/L, and is in accordance with the pressure drop increase observed in the PHE. As assumed earlier, the structure of the fouling layer becomes progressively different (more spaces and porosity), and this less compact structure is in ability to counterbalance the higher thickness of the deposit, allowing probably to maintain heat transfer value at a similar level.

The fact that Ca^2+^ is a vital element facilitating the build-up of the fouling layer has already been reported but in absence of P. Indeed, Khaldi et al. [8] showed that for the samples with high Ca/β-lg ratio, the Ca and S are homogeneously dispatched and co-located forming a thick layer. On the other hand, for low Ca/β-lg ratio, the layers were thin and compact since free ionic calcium content can no longer participate to the formation of the fouling layer and the fouling growth is only fed by protein-protein interactions (here protein involved is β-lg).

In the present work, for P concentration higher than 20 mg/L, the fouling growth ceases to be formed by Ca mediation and starts probably by CaP clusters (overlay images, Figure 7), which are not present in Khaldi et al. study.

### 3.3. Effect of Adding Phosphate on the β-Lg Denaturation Kinetics and Highlights on Fouling Build-Up in Function of Unfolding and Aggregation Reactions

As described elsewhere [36,40], Ca^2+^ content changes the β-lg denaturation kinetics. As phosphorus binds to Ca^2+^ changing Ca^2+^ concentration of the fouling solution, it is supposed that adding P to a WPI solution will affect the β-lg denaturation kinetics. However, no kinetics data are available in literature. To verify this assumption, β-lg denaturation kinetics was performed in the WPI solution with different phosphorus contents.

**Figure 8:**
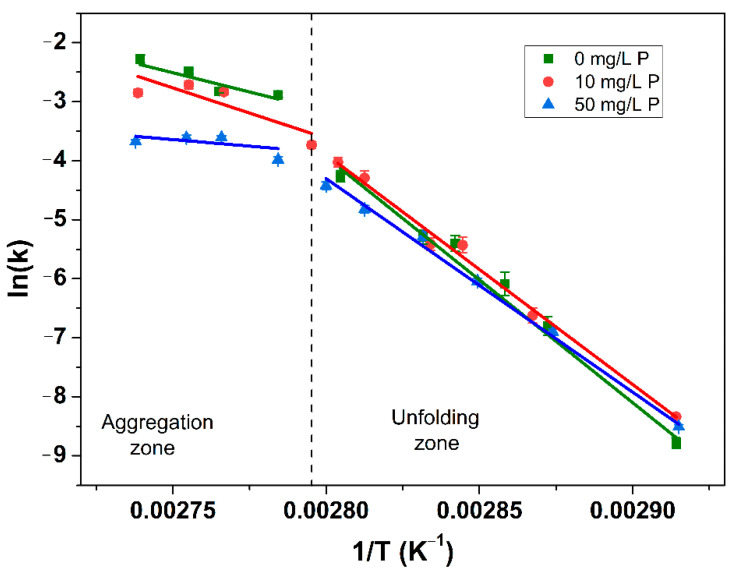
Arrhenius plot for the β-lg denaturation reaction with reaction order of 1.5 at various P concentrations. The solid lines correspond to the linear regressions of experimental data and the vertical dotted line correspond to 85 °C.

Figure 8 represents the Arrhenius plot of the denaturation reaction of β-lg at three added P concentrations (0, 10, and 50 mg/L) for a WPI solution with a Ca/β-lg molar ratio of 15.

For each P content, it can be observed that the denaturation constant (k) decreases with the inverse of temperature (T). However, the dependence of k with T cannot be described by a unique Arrhenius equation for the whole range of temperature once a sharp bend appears around 86 °C, whatever the added P concentration.

Analysis of Figure 8 reveals that the shape of the denaturation curve obtained in presence of added P (circle and triangular symbols) are not so different than the one obtained in absence of P (square symbols).

Otherwise, this shape is coherent with denaturation kinetics obtained for whey protein solutions containing mainly free Ca with different components concentrations (lactose, fat, protein, calcium) [8,35,41]. The critical temperature noticed for the break of slop (86 °C) is also in accordance with those reported in the literature for whey protein solutions without added P. Indeed, these studies report a critical temperature between 85 °C and 95 °C [34,42,43,44,45].

It is widely described in the literature that the sharp bend reveals the existence of two successive kinetically-driven reactions [35,44]: unfolding and aggregation of β-lg. Below 86 °C, the β-lg denaturation reaction is unfolding limited while over 86 °C, the aggregation is the limiting reaction. The frequency factor logarithm, ln(k_0_), and activation energies, E_A_, obtained by fitting Arrhenius curves, Equation (10) for the unfolding (unf) and aggregation (agg) zone, are shown in Table 1. Once again, the calculated parameters are also not so different with those reported in the literature [8,26,35,44] for other derivative whey protein solutions in absence of P. It is difficult to comment further since no values of these thermodynamic parameters are available, in our knowledge, for WPI solutions in presence of calcium chelator agents, such as phosphorus used in this work.

Further analysis of Figure 8 reveals that adding P in the WPI solution, induce a shift of denaturation reaction rate, ln(k). This shift due to the increase of added P concentration is clearly higher for aggregation zone compared to unfolding zone. These differentiate shifts indicate us that the addition of phosphorus has less impact on the apparition of reactive foulant species (unfolded protein) than on the aggregation of β-lg molecules.

This kinetic curves describing, at a molecular level, the general trends followed by unfolding and aggregation reactions in the bulk for a large range of temperature are interesting since they explained the results obtained at pilot scale when whey proteins solutions with and without chelating agents was heat-treated from 60 °C to 85 °C. Namely, high and small differences are observed concerning the non-aggregated species measured by HPLC on liquid samples collected at the outlet of the PHE for the solution containing respectively 50 mg/L and 10 mg/L of added P, in comparison with solutions without P, Table 2.

Without a big difference in the unfolded protein amount, one could expect the same deposit mass in the PHE surface, once the deposition fouling rate is governed mainly by the unfolded species concentration [46], which was not the case, Figure 2. This confirms the fact that the protein reactivity in the heating zone does not depend on the kinetics of release of unfolded proteins. The decrease in the fouling mass for 50 mg/L of added P, Figure 2, should, therefore, be explained by the reduction of the aggregation ability of unfolded protein when 50 mg/L of P were added.

Indeed, if the fouling is limited, it is not due to a change in reactive material but to other phenomena, such as the decrease in free calcium concentration, which binds the strands of unfolded proteins. This element clearly shows that a change in the fouling mechanism is induced, no doubt due to the lack of binding agents that was transformed in CaP, in presence of 50 mg/L.

## 4. Conclusions

In this paper, the addition of disodium hydrogen phosphate (Na_2_HPO_4_) in a whey protein isolate solution was used to demonstrate how depletion of calcium ions affects fouling mechanisms during a pasteurization heat-treatment.

Depending on the level of added P in the WPI solution (i.e., depletion of calcium ion achieved), it is shown that the fouling growth on the PHE can happen, according to two different mechanisms.

For P concentrations lower than 20 mg/L, the total fouling mass and the heat transfer coefficient were kept almost constant, and the pressure drop increased. For concentrations of added P, Ca was in excess in the WPI solution and the addition of P contributed only to chelate a part of the Ca^2+^, but this chelation is not exhaustive enough to change the fouling layer composition, which is mainly based on co-located calcium and protein. However, the increase in pressure drop with P concentration suggests that the fouling deposit layer becomes progressively less compact and gives rise to a more voluminous protein/mineral deposit without any contribution of CaP clusters as indicated by EPMA. At a molecular level, it is assumed that the protein charge screening effect by Ca^2+^ is reduced, favoring repulsion between proteins inside the deposit and, thus, resulting in a more open structure.

For a higher P concentration (>20 mg/L), the total fouling mass starts to decrease while the pressure drop and the heat transfer coefficient remain almost constant during the whole running time. From this P concentration, there is enough phosphorus in the solution to chelate calcium forming CaP clusters, as showed by EPMA overlay images. The fouling layer composed of CaP clusters surrounded by proteins forms a non-homogeneously distributed arborescent structure. Furthermore, the fouling build-up starts from a CaP layer, which is concentrated close to the steel surface. At a molecular level, the presence of a CaP cluster is a tangible indicator that the Ca^2+^ concentration has reached a minimum value and so it is expected that the interactions between protein and free calcium can no longer occur and, consequently, protein species and calcium elements can only initiate association through CaP–protein interactions. As all of the CaP are on the steel surface, there is little CaP on solution to continue the fouling build-up. This mean that, in this case, the fouling growth is not limited by the lack of reactive protein, but by the lack of binding agent.

Due to the different fouling mechanism and structure in function of the added P concentration, it is also expected that the cleaning parameters (temperature, alkali/acid composition, and duration) might also change.

## Figures and Tables

**Table 1:** Calculated denaturation parameters at various P concentration.

	P Concentration (mg/L)		
Denaturation Parameter	0	10	50
Unfolding			
E_Aunf_ (kJ/mol)	343.3	327.9	296.5
ln(k^o^_unf_)	111.7	106.6	95.5
Aggregation			
E_Aagg_ (kJ/mol)	119.2	140.1	52.6
ln(k^o^_agg_)	37.0	43.5	13.8

**Table 2:** Measured initial (inlet of the PHE) and final (outlet of the PHE) soluble β-lg (non-aggregated) concentration.

P Concentration (mg/L)	Non-Aggregated Protein Concentration (g/L)		Aggregation Level (%)
	Inlet (60 °C)	Outlet (85 °C)	
0	3.13	0.97	69
10	3.07	0.96	69
50	3.08	2.21	28
